# How Does Food Taste in Anorexia and Bulimia Nervosa? A Protocol for a Quasi-Experimental, Cross-Sectional Design to Investigate Taste Aversion or Increased Hedonic Valence of Food in Eating Disorders

**DOI:** 10.3389/fpsyg.2018.00264

**Published:** 2018-03-13

**Authors:** David Garcia-Burgos, Sabine Maglieri, Claus Vögele, Simone Munsch

**Affiliations:** ^1^Department of Psychology, University of Fribourg, Fribourg, Switzerland; ^2^School of Agricultural, Forest and Food Sciences HAFL, Bern University of Applied Sciences, Bern, Switzerland; ^3^Institute for Health and Behaviour, Research Unit INSIDE, University of Luxembourg, Esch-sur-Alzette, Luxembourg

**Keywords:** eating disorders, food avoidance, hedonics of taste, signal detection theory, taste aversion

## Abstract

**Background:** Despite on-going efforts to better understand dysregulated eating, the olfactory-gustatory deficits and food preferences in eating disorders (ED), and the mechanisms underlying the perception of and responses to food properties in anorexia nervosa (AN) and bulimia nervosa (BN) remain largely unknown; both during the course of the illness and compared to healthy populations. It is, therefore, necessary to systematically investigate the gustatory perception and hedonics of taste in patients with AN and BN. To this end, we will examine whether aversions to the taste of high-calorie food is related to the suppression of energy intake in restricting-type AN, and whether an increased hedonic valence of sweet, caloric-dense foods may be part of the mechanisms triggering binge-eating episodes in BN. In addition, the role of cognitions influencing these mechanisms will be examined.

**Method:** In study 1, four mixtures of sweet-fat stimuli will be presented in a sensory two-alternative forced-choice test involving signal detection analysis. In study 2, a full-scale taste reactivity test will be carried out, including psychophysiological and behavioral measures to assess subtle and covert hedonic changes. We will compare the responses of currently-ill AN and BN patients to those who have recovered from AN and BN, and also to those of healthy normal-weight and underweight individuals without any eating disorder pathology.

**Discussion:** If taste response profiles are differentially linked to ED types, then future studies should investigate whether taste responsiveness represents a useful diagnostic measure in the prevention, assessment and treatment of EDs. The expected results on cognitive mechanisms in the top-down processes of food hedonics will complement current models and contribute to the refinement of interventions to change cognitive aspects of taste aversions, to establish functional food preferences and to better manage food cravings associated with binge-eating episodes. No trial registration was required for this protocol, which was approved by the Swiss ethics committee (CER-VD, n° 2016-02150) and the Ethics Review Panel of the University of Luxembourg.

## Background

Anorexia nervosa (AN) and bulimia nervosa (BN) are serious, often treatment-refractory mental illnesses, including medical complications, frequent comorbidity, and high mortality (Westmoreland et al., 2016). Despite extensive research in eating disorders (EDs), the core phenomenon of extremes in appetite (too little or too much) and the perception of food taste remains scarcely investigated. This is surprising as the perception of food plays an important role in the maintenance of disordered eating behaviors (e.g., restrictive food intake, binge-eating of palatable food) and ED interventions (e.g., targeting forbidden foods, unbalanced diet). This research aims to shed light on the gustatory and taste hedonic responses to high-calorie food in AN (restrictive-type) compared to BN and healthy individuals, and how they are biased by cognitions of gaining weight. The novel and most critical assumption of our approach is that the effect of food on eating behavior arises actively through perceptual processes (i.e., within the brain), not just passively from physicochemical properties of the foods themselves (Berridge et al., 2010).

Previous research on gustometry have found decreased or altered taste sensitivity and hypogeusia in AN and BN patients (Casper et al., 1980; Drewnowski et al., 1987b; Nakai et al., 1987; Aschenbrenner et al., 2008), which are often assumed to facilitate self-starvation via decreased taste qualities of feeding in EDs. Factors known to affect taste perception in individuals with EDs include the reduced number of fungiform papillae on the tongue in AN and damages in the palate caused by vomiting in BN (Rodin et al., 1990; Wöckel et al., 2007). These findings, however, remain controversial as other studies have not found such abnormalities (Di Costanzo et al., 1998; Vocks et al., 2011; Goldzak-Kunik et al., 2012; Dazzi et al., 2013), and deficits seem to be rather a consequence than a cause of low body weight, metabolic abnormalities, duration of the illness or malnutrition. Gustatory alterations are partially reversible after weight restoration (Aschenbrenner et al., 2008), and amenable to psychological interventions (Goldzak-Kunik et al., 2012). For example, Nozoe et al. (1996) found taste responsiveness in AN to improve after behavior therapy, even prior to any increase in body weight. AN patients have also been shown to respond differently to swallowed than expectorated sugar solutions, suggesting that responses are driven by the expectancy of calories rather than the taste *per-se* (Eiber et al., 2002). Thus, cognitions concerning the fear of weight gain are likely to affect the experience of taste perception, closing a pathological circle, which maintains the core symptoms of AN (Monje Moreno et al., 2014).

Overall, gustatory experiences are reported by BN patients as less intense compared with healthy controls, though the average scores are within the range of normogeusia (Nakai et al., 1987; Jirik-Babb and Katz, 1988). When flavors are applied to the tongue instead of sipped (e.g., using the taste strip methodology), lowered gustatory sensitivities have been found (Rodin et al., 1990; Aschenbrenner et al., 2008; Dazzi et al., 2013) in BN patients. As whole-mouth taste experience is not altered and only a few studies with mixed results have examined the gustatory response in BN, it remains unclear whether these alterations are a consequence or a predisposing factor for BN.

Importantly, taste sensations elicit strong affective^1^ responses (Vögele et al., 2018). While altered hedonic reactions to pleasant-tasting food are a feature of AN, it is unclear whether this is due to increased disliking of high-caloric products, or reduced liking associated with low sensitivity to food pleasantness. The current evidence does not point to a deficit in experiencing taste hedonics in AN, as self-report measures show similar responses to low-energy foods, aversive tastes or sucrose solutions in the sip-and-spit paradigm compared to controls (Eiber et al., 2002; Jiang et al., 2010; Szalay et al., 2010). Rather, individuals diagnosed with AN show enhanced dislike for the taste of high-energy foods (Drewnowski et al., 1987b; Sunday and Halmi, 1990; Stoner et al., 1996; Tóth et al., 2004), especially those with restrictive-type AN (Cowdrey et al., 2013). Perhaps even more important, hedonic ratings tend to be strongly reduced by an overriding fear of weight gain in paradigms where sucrose solutions are swallowed compared to when these solutions are spat out (Eiber et al., 2002). The use of self-report measures for the investigation of hedonics in individuals with AN, however, is limited as they are often characterized by difficulties in detecting and describing their own and others emotions (Bydlowski et al., 2005; Vögele et al., 2018). Therefore, objective measures (i.e., those less prone to be affected by conscious control) to assess hedonic reactions to taste (e.g., facial responses) are needed. To the best of our knowledge, only three studies have applied either facial electromyographic activity (Friederich et al., 2006; Soussignan et al., 2011) or orofacial expression analysis (Szalay et al., 2010) to assess taste hedonics in AN. Together, these studies suggest reduced automatic pleasantness processing and weaker reactivity in AN compared to non-eating disordered controls. Nevertheless, these results remain inconclusive, as other potential mediators of food hedonics (e.g., exercise, depressive mood, hunger, satiety states, and food reward sensitivity) were not considered.

In BN patients, results on taste hedonics are inconsistent and vary with respect to the dimensions of calorie content (high vs. low), taste (fat vs. sweet) and history of AN. While both AN and BN patients avoid fat taste, only bulimics without a history of AN show higher liking ratings for high-sweet taste stimuli compared to controls (Garner et al., 1978; Drewnowski et al., 1987a; Sunday and Halmi, 1990; Franko et al., 1994), but without differences for low-sweet taste solutions (Rodin et al., 1990). Interestingly, compared with restrictive AN and healthy controls, a greater proportion of BN women (and binge-eating/purging AN) reported use of artificially sweetened low-calorie products and “patients described consuming the sweetener directly from the packet, using 100 or more packets daily” (Klein et al., 2006, p. 344), suggesting an increased drive for sweet orosensory stimulation. Moreover, during binges, BN individuals tend to eat high-caloric foods they would otherwise avoid, and their primary choice of foods is high in carbohydrates (van der Ster Wallin et al., 1994). Whether binge eating is characterized by craving for those very sweet stimuli remains largely unexplored.

**Figure 1:**
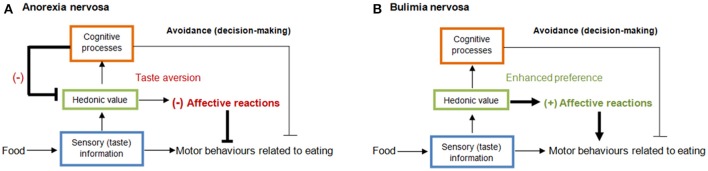
Serial hierarchical processing of taste (Wolz et al., 2015) at the sensory (blue), reward/hedonic (green), and cognitive level (red) via bottom-up influences. In restricting AN **(A)**, biased cognitions about body weight (e.g., “this food increases body weight”) make one feel bad while eating and these negative feelings extend to the affective value of taste (e.g., disgust; Bernstein and Borson, 1986) making caloric food taste worse (taste aversion) and ceasing intake. It is worth noting that cognitions can reach down into the taste system in the orbitofrontal cortex, which controls how pleasant a taste stimulus is (Grabenhorst et al., 2008; Rolls, 2015). In BN **(B)**, there is a motivational conflict between avoiding food because of anticipation of unpleasant feelings underlying becoming fat and the enhanced pleasant reactions to sweet taste, which finally override the inhibitory cognitive control to resist food craving and exacerbate food consumption, and thus binge-eating episodes. (–) aversive or (+) appetitive affective activation.

In summary, these findings suggest that taste disturbances in AN and BN are rather a consequence than the cause of the respective ED. It remains unclear, however, whether these disturbances are a result of the physical (e.g., body mass index [BMI], physiological dysfunction) or the psychological changes associated with AN/BN (e.g., biased cognitions, mood, depressive mood). Concerning taste hedonics, it is important to better understand the processes motivating approach/avoidance behavior with regards to low- and high-calorie foods (Cowdrey et al., 2013; Wolz et al., 2015). Based on the current literature, we would argue that the perception of calorie intake and the fear of weight gain are responsible for the avoidance of high-caloric food in AN and BN, mainly through two mechanisms: a first mechanism (more intentional) is based on resisting food craving actions in BN (who indeed like high-sweet food) and another (more visceral) is based on learned taste aversion in those individuals with restricting-type AN (Bernstein and Borson, 1986), who respond to palatable food as if its taste were disgusting (Chapman and Anderson, 2012). An explanation about how the mechanisms contribute to caloric restriction and binge eating is offered in Figure 1. The main aim of the present research, therefore, is to systematically investigate the gustatory perception and hedonics of taste. We will examine whether aversions to the taste of high-calorie food is related to the suppression of energy intake in restricting-type AN, and whether an increased hedonic valence of sweet, caloric-dense foods may be part of the mechanisms triggering binge-eating episodes in BN. We will also examine the role of cognitions influencing these mechanisms.

Hypothesis

H1: If perceptual disturbances in AN and BN are related to biased cognitions about gaining weight rather than to gustatory deficits or BMI, then AN and BN patients should respond with similar sensory performance but higher response bias scores in a discrimination test, (1) compared to non-ED underweight, normal-weight and illness-recovered controls, and (2) during a swallowing compared with a sip-and-spit condition.
H2: If fear of weight-gain in restricting-type AN elicits taste aversion, then facial electromyographical, facial expression, and autonomic and subjective correlates of disgust (Kappas, 2003; Kreibig, 2010) while tasting high-sweet and high-fat foods should be observed compared to (1) stimuli of low-sweet and low-fat foods; (2) non-ED underweight, normal-weight, illness-recovered controls and (3) BN patients; and (4) especially during the swallowing compared with the sip-and-spit condition.
H3: If avoidance of energy-rich foods in BN reflects a cognitive preoccupation about the increased liking of very sweet tastes associated with gaining weight, then facial electromyographical, facial expression, and autonomic and subjective correlates of pleasure (Kappas, 2003; Kreibig, 2010) while tasting high-sweet foods should be observed compared to (1) fat and low-sweet stimuli; (2) non-ED underweight, normal-weight, illness-recovered controls and (3) restrictive-type AN; and (4) no differences between swallowing and sip-and-spit conditions.

## Methods

### Participants

A total sample of 160 female participants will be recruited in Switzerland and Luxembourg. AN (restricting-type), BN (without history of AN) patients meeting DSM-5 (American Psychiatric Association, 2013) criteria will be divided into currently-ill (C) and recovered (R). Thus, C-AN (*n* = 30) and C-BN (*n* = 30) will be compared to R-AN (*n* = 20) and R-BN (*n* = 20) patients, as well as to two age-matched control (CT) groups: underweight (U-CT; *n* = 30) and normal-weight (N-CT; *n* = 30) without eating disorders. BN with previous history of AN or binge-eating/purging type of AN will be excluded to enable a better differentiation between these disorders. Participants will be asked to provide demographic information, height, weight, duration of illness, time in treatment, age at onset, duration of recovery where applicable, and self-reported salivary, metabolic, and/or otorhinolaryngologic disorders. Ethical permission was obtained from Swiss Ethics Committee and from the Ethics Review Panel of the University of Luxembourg. Participants will provide written informed consent.

### Inclusion and exclusion criteria

Principal inclusion and exclusion criteria are shown in Table 1.

**Table 1:** Principal inclusion and exclusion criteria.

**INCLUSION CRITERIA**
**Normal and underweight control groups**
Female sex
Age from 18 to 35 years of age
Informed consent with understanding of the study and procedures
No eating disorder history
BMI < 18 (underweight subgroup) or 18.5 < BMI < 25 (normal-weight subgroup)
**Current anorexia and bulimia nervosa groups**
Diagnoses of AN (restricting-type without previous history of binge-eating/purging type) or BN (without history of AN)
If treatment has started, reduction of the initial eating disorder pathology measured by EDE-Q (Mond et al., 2004) <30%.
<50% of the target weight gain (AN) or <30% reduction of binge eating and compensatory episodes (BN)
**Recovered anorexia and bulimia nervosa groups**
Not meeting all criteria for an AN or BN at the time of discharge
BMI > 18 and a global EDE-Q-score <2.3 (Munsch, 2014)
**EXCLUSION CRITERIA**
Pregnancy or lactation
Psychotic and related disorders or depressive disorders*
Serious medical conditions having an effect on eating and mood
Lack of compliance with study procedure
Past bariatric surgery
Allergy for the foods offered
History of olfactory or gustatory pathology, and salivary, metabolic or otorhinolaryngologic disorders (e.g., ageusia, dysgeusia, anosmia, hyposmia, allergic rhinitis, chronic rhinosinusitis, and upper respiratory infection)

Smoking (more than 5 cigarettes per week) and current intake of serotonin-specific reuptake inhibitors will not be initially considered an exclusion criterion, but will be recorded carefully. Phenylketonuric people will be appropriately warned as the high sweet samples content Aspartam, a source of Phenylalanin. *If they prevent a participation in the trial.

### Ethical aspects and approval

The study was approved by the ethical committee of the leading center at the University of Fribourg (CER-VD, n° 2016-02150) as well as in the cantons of collaborating clinics in Switzerland and the Ethics Review Panel of the University of Luxembourg. Written informed consent in accordance with the Declaration of Helsinki will be obtained (Declaration of Helsinki). All procedures within this research project will be conducted in accordance with the guidelines for Good Clinical Practice by clinically trained investigators under the permanent supervision of the main applicants. All data will be coded without personal identifiers to ensure confidentiality. Participants may withdraw from the trial at any point without any penalty.

### Procedure: recruitment, sample characterization, and experimental session

**Figure 2:**
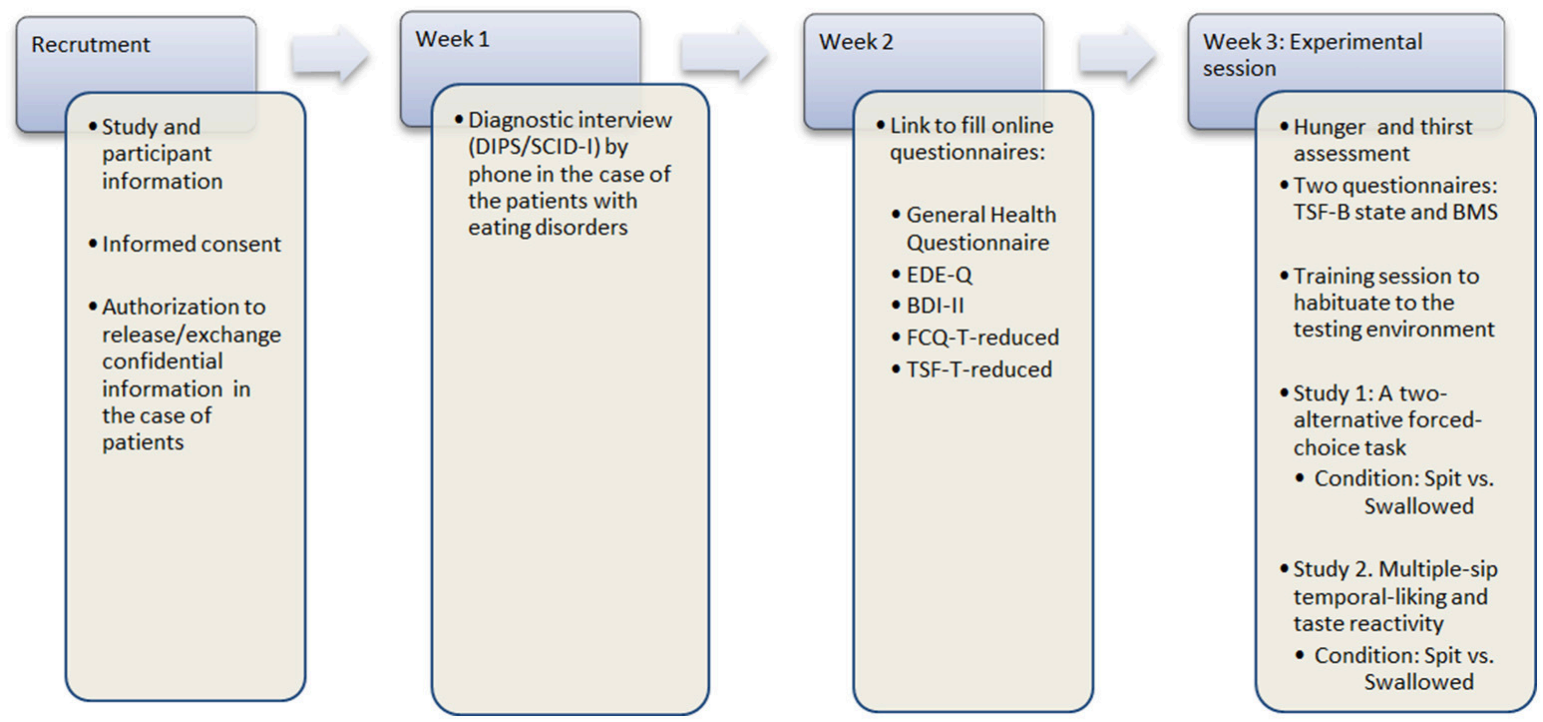
Flow graph of the procedure.

The procedure is summarized in Figure 2. The participant recruitment process starts with a recruitment letter to inform female patients between 18 and 35 years of age, starting or finishing with treatment, about our study at the collaborating clinics or with advertising flyers in public places and at the University in the case of healthy control participants. If the patient agrees, and after obtaining informed consent and the authorization to release/exchange confidential information, they will be contacted by phone to set three appointments in weekly intervals. During the first week, a diagnostic interview will be carried out by telephone. Eligible participants will be required to fill out a set of online questionnaires (LimeSurvey; www.limesurvey.org), including the General Health Questionnaire, EDE-Q, short form of the Beck Depression Inventory-II (BDI-II), Food Cravings Questionnaire—Trait (FCQ-T-reduced) and Thought-Shape Fusion Questionnaire (TSF-Trait) (see Table 2) during the second week. Finally, the experimental session in the clinics or in the psychophysiological laboratory at the universities of Fribourg and Luxembourg are expected in the third week. In the case of healthy controls, the procedure will be the same except for the diagnostic interview.

**Table 2:** Summary of the measures, instruments and outcomes.

**Type**	**Measure/instrument**	**Measure description**	**Device/author**	**Outcome**	**Study**
Subjective	Diagnostic interview for psychiatric disorders (**Mini-DIPS**) and Structured Clinical Interview for DSM-IV (**SCID-I**)	Structured interviews to assess psychiatric disorders according DSM-IVTR and DSM-5	Wittchen et al., 1997; Schneider and Margraf, 2011	Diagnostic phase	Study 1 & 2
	Eating Disorder Examination Questionnaire (**EDE-Q**) (score)	36 items scored on a 7-point Likert-type scale reporting restraint, eating concerns, weight concerns and shape concerns, as well as frequencies of compensatory behaviors are also self-reported. Internal consistency >0.70; test-retest reliability >0.50	Mond et al., 2004	Diagnostic phase	Study 1 & 2
	Thought-Shape Fusion Body State Scale (**TSF-B state**)	5-item questionnaire assessing body-related cognitive distortions according to Radomsky et al., 2002. Each item rated on a 100-mm VAS scale (0–100). Cronbach's alpha = 0.86	Wyssen et al., 2016	Baseline	Study 1 & 2
	Beck Depression Inventory-II (**BDI-II**) (score)	21 items rated in a 3-point scale indicates depression raging from normal mood to severe depression. Internal consistency >0.90; test-retest reliability >0.90; convergent validity >0.71	Beck et al., 1996	Baseline	Study 1 & 2
	Food Cravings Questionnaire—Trait (**FCQ-T-reduced**) (score)	15 items scored on a 6-point scale related with eating pathology, body mass index, low dieting success and increases in state food craving during cognitive tasks involving appealing food stimuli. Internal consistency >0.70; validity demonstrated by positive correlations between scores on the FCQ-T-r, BMI and cue-elicited FC; convergent validity >0.50	Meule et al., 2011	Baseline	Study 1 & 2
	Thought-Shape Fusion Questionnaire (**TSF-Trait**) (score)	18 items which measures the susceptibility to body-related cognitive distortions	Coelho et al., 2013; Wyssen et al., in press	Baseline	Study 1 & 2
	The Brief Mood Scale (**BMS**)	8 bipolar items on a 100-mm VAS (0–100). Cronbach's alpha >0.88	Wilhelm and Schoebi, 2007	Baseline	Study 1 & 2
	**Ratings**: sweetness and fattiness identity and intensity (score) **SDT parameters**: d′ (sensory sensitiviy), C and ß (response bias based on cognitive strategy)	11-point anchored Likert-type scale (scores from 0 to 10) designed to measure taste perception	Sussex Ingestion Pattern Monitor (SIPM™ 2.0); Sussex University; UK	Primary	Study 1
	**Ratings**: pleasantness of the taste (score)/thirst and hunger level (score)/familiarity of food samples.	Time-intensity measures for pleasure; opportunity to scale the perceived liking dynamically over 60 sec. Labeled magnitude scale for thirst/hunger	SensoMaker™; UFLA, Brazil	Primary/secondary	Study 1 & 2
Behavioural	**Facial expressions**: general emotions, valence and actions units (intensity score)	Positive/negative valence as measure of overall affection; general emotions (joy, anger, surprise, fear, contempt, sadness, disgust); action units as the likelihood of specific facial muscle activations	FACET™ SDK; iMotions Inc., Cambridge Innovation Center, US	Primary	Study 1 & 2
Psycho-physiological	**Cardiovascular**: heart rate and heart rate variability	Cardiovascular Changes as index of cardiac sympathetic activation and autonomic arousal	Module—GSR & Heart Rate and ECG/EMG; Shimmer3 EMG/ECG, GSR Kit and EXG Devices; iMotions Inc., Cambridge Innovation Center, US	Primary	Study 2
	**Electrodermal activity**: skin conductance level (μmhos), skin conductance response rate (count/min)	Changes in the conductivity of skin as index of sympathetic activation		Primary	Study 2
	**Facial electromyography**: EMG powers (mV)	EMG activity of the levator labii (disgust), corrugator supercilia (general negative affect) and zygomatic major (pleasure) muscle regions		Primary	Study 2

The experimental session will be composed of assessments of thirst and hunger levels, the Thought-Shape Fusion Body State Scale (TSF-B state) and Brief Mood Scale (BMS) questionnaire, a training session to habituate to the testing environment and apparatus, and either the discrimination of two-alternative forced-choice (2AFC) test in study 1 or multiple-sip temporal-liking (MSTL) with taste reactivity (TR) task in study 2. Each experimental session will take around 1.5 h. Concerning the first study (Table 3), the 2AFC test with signal detection theory (SDT) analysis will be used as it provides a high level of power for small food differences and low levels of response bias (Hautus et al., 2009). On the one hand, the 2AFC test is a psychophysical method in which participants are required to select the one of two food samples, which represents the specified attribute best (e.g., the sweeter one). On the other hand, SDT is a sophisticated tool that permits to understand the information processing that takes place in the brain during sensory discrimination testing, providing an accurate index of perceived sensory differences and efficient characterization of cognitive strategy. Therefore, SDT is critical for investigating maladaptive cognitions involved in the processing of received taste information, which affects sensory testing (Smeets et al., 2008). Furthermore, whether and how much gustatory performance depends on low BMI or psychopathology will be examined including under- and normal-weight controls and patients at different stages of the disorder (currently-ill vs. recovered). Finally, in addition to the sip-and-spit condition, a swallow test condition will be included (Eiber et al., 2002) to enhance cognitive bias toward weigh gain underlying the fear of swallowing and quantify the impact of these biased cognitions on gustatory performance. The 2AFC task will be conducted using the SIPM™ software (Sussex Ingestion Pattern Monitoring, version 2.0.11), in which pairs of two samples (with varying levels of sweetness and fatness), presented simultaneously at each trial, are judged in terms of taste identity and intensity (see Table 2).

**Table 3:** Experimental design of the study 1.

**Group**	**Pre-test**	**Test: two-alternative forced-choice task**	
		**Spit condition**	**Swallowed condition**
R-AN	Training session to habituate to the testing environment	A vs. B, A vs. C, A vs. D, B vs. C, B vs. D, C vs. D	A vs. B, A vs. C, A vs. D, B vs. C, B vs. D, C vs. D
C-AN			
U-CT			
N-CT			
R-BN			
C-BN			

*A, B, C, D, samples of ice cream with a high-/low-sweet and high-/low-fat taste. The order of the paired samples and conditions (swallow and spit) will be randomized. R-AN, recovered anorectic; C-AN, currently ill anorectic; U-CT, underweight control; N-CT, normal weight control; R-BN, recovered bulimic; C-BN, currently ill bulimic. R-AN and R-BN will be assessed at discharge*.

Concerning the second study (Table 4), a full-scale hedonic taste methodology using MSTL with TR will be applied to provide self-report, facial expression, facial electromyographic and autonomic measures to investigate (1) whether restrictive-type AN individuals respond with taste aversion for high-calorie foods and (2) if individuals with BN respond with excessive pleasure to sweet tastes. The MSTL and TR methodologies have been successfully employed previously with healthy populations (Rocha-Parra et al., 2016), showing both to be highly sensitive to detect small emotional variations when hedonic reactions are monitored dynamically via cumulative measurements, multiples sips and measurements at different durations. Consequently, this approach will allow for addressing two important unresolved issues: the temporal aspects of the hedonic response while eating (Delarue and Blumenthal, 2015), which have received no attention in EDs despite clinically observable aberrant emotional processing, and the scarce data on taste reactivity in EDs, which is considered the gold standard measure of hedonic aspect of human gustation without being limited by cognitive appraisal confounders of self-reports. MSTL, in which participants report subjective hedonic evaluations with SensoMaker™ software (version 1.8; Pinheiro et al., 2013) during 60 s while drinking three sips of each sample, taking one sip every 20 s, and TR measures (facial expression, facial electromyographic, electrodermal and heart rate reactivity) (Table 2) will be recorded while tasting food samples. The video files will be run through the FACET™ SDK (iMotions Inc., Cambridge Innovation Center, US), which is an automatic facial expression recognition software that tracks and analyzes frame-by-frame (1/25 s) valence, action units and emotions. Finally, the motivational component (i.e., desire to eat) of hedonic reward of each food sample will be also assessed at the end of the 60-s period, asking “How much do you want to eat this food?” Participants' responses will be monitored using a 20 cm unstructured line scale anchored at both extremes 0–100 on the monitor (0 = “not at all”; 100 = “extremely”). Finally, in order to even increase patients' safety and well-being the standardized procedure in clinics will be enlarged by a short muscle relaxation training session of 15 min offered to patients at the end of the experimental session for study 1/study 2 to reduce any anxiety raised by tasting chocolate ice cream samples.

**Table 4:** Experimental design of the study 2.

**Group**	**Pre-test**	**Test: multiple-sip temporal-liking and taste reactivity**	
		**Spit condition**	**Swallowed condition**
R-AN	Training session to habituate to the testing environment	3 sips × water, 3 sips × A, 3 sips × B, 3 sips × C, 3 sips × D, 3 sips × E, 3 sips × F	3 sips × water, 3 sips × A, 3 sips × B, 3 sips × C, 3 sips × D, 3 sips × E, 3 sips × F
C-AN			
U-CT			
N-CT			
R-BN			
C-BN			
			

*A, B, C, D, samples of ice cream with a high-/low-sweet and high-/low-fat taste. E, F, samples of savory high-fat and low-fat sauces, respectively; R-AN, recovered anorectic; C-AN, currently ill anorectic; U-CT, underweight control; N-CT, normal weight control; R-BN, recovered bulimic; C-BN, currently ill bulimic. R-AN and R-BN will be assessed at discharge*.

### Food samples

In contrast to pure tastes commonly used in research on gustatory perception, four ordinary, more ecologically valid mixtures of chocolate ice cream containing low vs. high levels of fatness and low vs. high levels of sweetness (Table 5) will be presented in 100-ml disposable Styrofoam cups at room temperature and in counterbalanced order across participants. Chocolate ice cream was selected according to the assumptions that chocolate ice cream is likely to be a forbidden food for AN and BN participants, and that AN participants can be expected to have strong aversive associations. The samples are stored at minus 18°C. Optimal mixtures were elaborated at the School of Agricultural, Forest and Food Sciences (Department of Food Science & Management; Bern University of Applied Sciences) and obtained considering previous studies on taste sensory and hedonic ratings (Sunday and Halmi, 1990) and surface response method (Drewnowski et al., 1987b) in AN and BN. Assuming an all-or-nothing (i.e., dichotomous) approach to food in AN and BN, regardless of the fat or sucrose levels, the specific ingredients of the samples were further selected in order to increase differences across ice cream samples in terms of sweetness, creaminess, expectation of caloric content and hedonic reactions (Table 6). In this sense, although previous studies have found pronounced aversion to the sugar-fat mixtures in AN patients, variability in aversive scores across combinations of sweet taste and fat together has also been demonstrated. For instance, differences comparing liking response profiles as a function of increasing sucrose content (0–20% wt/wt) separately from different fat samples (0.1–52.7% fat wt/wt) have been observed in AN (restrictive) and bulimic patients, and in both currently-ill and recovered patients (e.g., Drewnowski et al., 1987b; Sunday and Halmi, 1990). Finally, while water will be used as control stimulus, two savory (high-fat and low-fat) sauces will be added in study 2 as additional comparators to demonstrate that any differences detected are unique to sweet-fat foods in BN patients as previous research suggests.

**Table 5:** Characterization of the food samples with low and high content of fatness and sweetness.

**Components**	**Low fatness and low sweetness**	**Low fatness and high sweetness**	**High fatness and low sweetness**	**High fatness and high sweetness**
Dry matter (%)	30.0	30.0	39.5	39.5
Fat content (%)	1.2	1.2	11.4	11.4
Carbohydrate (g)	21.8	21.8	22.1	22.1
Protein (g)	4.7	4.8	4.8	4.8
Fat (g)	1.2	1.2	11.9	11.9
Kcal total	117.0	117.3	215.1	215.4
Kcal (%)	54.3	54.4	99.9	Reference = 100
Relative sweetness	14.45	28.01	14.76	28.8
**INGREDIENTS (%)**				
Whole milk	0.0	0.0	35.0	35.0
Skimmed milk	45.6	45.6	0.0	0.0
Cream	0.0	0.0	27.2	27.2
Water	27.1	27.1	11.0	11.0
Skimmed milk powder	7.6	7.6	4.2	4.2
Sucrose	8.7	8.7	6.8	6.8
Dextrose	4.3	4.3	4.5	4.5
Glucose DE 35–40%	2.2	2.2	1.5	1.5
Sweetener (Aspartam)	0.0	0.1	0.0	0.1
Stabilisator/emulfiser	0.8	0.8	0.7	0.7
Chocolate powder	0.9	0.9	0.8	0.8
Cocoa powder	2.8	2.8	2.6	2.6
Color (E150d)	0.6	0.6	2.0	2.0
Flavor	0.1	0.1	0.1	0.1
Polydextrose	0.0	0.0	4.5	4.5
Whey powder	0.0	0.0	2.0	2.0

*The high fatness and high sweetness is the food sample reference for kcal*.

**Table 6:** Preliminary sensory testing results (*N* = 5; normal weight individuals) for the food samples with low and high content of fatness and sweetness.

**Attribute**	**Food sample**			
	**Low fatness and low sweetness**	**Low fatness and high sweetness**	**High fatness and low sweetness**	**High fatness and high sweetness**
Sweetness	2.75	5.96	3.50	6.25
Creaminess	2.17	3.42	4.96	5.50
Chocolate flavor	4.08	4.58	4.17	4.63
Caloric content	3.00	5.83	5.21	6.50

*The attributes were rated in a scale from 1 (not sweet, not creamy, too week, very low) to 7 (very sweet, vey creamy, too intensive, very hing)*.

### Outcome measures

Information about the primary and additional secondary outcome measures and instruments is summarized in Table 2.

### Study design

The present international and multi-center research uses a quasi-experimental, cross-sectional design with concurrent controls aims at comparing gustatory perception and hedonics of taste among six groups of participants: R-AN, C-AN, U-CT, N-CT, R-BN, and C-BN, under two counterbalanced conditions of testing: sip-and-spit vs. swallow test condition. The use of a rigorous quasi-experimental design with a well-established type of psychopathology represents an important approach in the experimental psychopathology literature (Zvolensky et al., 2013), particularly in the context of eating disorders for both ethical and pragmatic reasons. However, the limitations are related to the lack of random assignment into groups, which leads to non-equal test groups, which can limit the generalizability of the results to a larger population. In order to reduce the impact of this limitation, threats to validity will be minimized controlling the following factors: comorbidity, stage-of-illness, age-at-onset, duration of illness and time in treatment, depressive mood, craving for food, biased cognitions about weight, current hunger, thirst and food familiarity. In addition, age-matched and weight-matched controls (to the extent possible) will be included in the research design to increase the chances of finding differences between groups. Additional controls by limiting our inclusion criteria are not envisaged as this would make it even more difficult to obtain samples of adequate size (therefore reducing statistical power) and limiting the generalizability (and hence the external validity) of the study protocol.

### Statistical and analytic plan

#### Sample size calculation

Sample size calculations were based on findings of small to medium effects on eating behavior in patients with anorexia and bulimia by our group (e.g., Munsch, 2014) using the software G^*^Power (version 3.13). Assuming two-sided tests with Alpha = 0.05, Beta = 0.2, and effect size *f* = 0.20, the required sample size would be 80 to ensure a power >0.80 in each one of the two studies with a sample distribution of N_C−AN_ = 15, N_C−BN_ = 15, N_R−AN_ = 10, N_R−BN_ = 10, N_U−CT_ = 15, and N_N−CT_ = 15. To obtain the required number of 50 patients per study and taking into account a participation rate of 80% and a dropout rate of 20% in the patient population, a total of 60 patients per study will be needed. Based on our recruitment and testing experiences in Switzerland and Luxembourg (average annual recruitment of 35–40 AN and 35–40 BN patients), the access to a sufficiently powered sample of patients seems to be guaranteed. Finally, in order to achieve the sample size suggested by the power analysis, a sample of 30 healthy controls (university students) per study will be recruited in Fribourg.

#### Data processing

In study 1, three direct measures will be derived from the response data of the 2AFC procedure using the SDT (Hautus et al., 2009): the unaffected sensory sensitivity measure (d′) and the two most popular measures of response bias (C and ß). Specifically, the SDT measures will be calculated following Stanislaw and Todorov's (1999) mathematical formulae, with d′ = [z(H)-z(F)]/√2 and z(H) and z(F) are transformations of the hit and false alarm rates to inverse z-scores; calculated in Excel as DPRIME = NORMSINV(H)-NORMSINV(F). Since the more sensitive is an individual the larger the value of d′, an individual who cannot discriminate levels of sweetness or fatness will have a d′ = 0. By contrast, the measure of response bias based directly on cognitive strategy will be calculated in Excel as C = −(NORMSINV(H)+NORMSINV(F))/2. Response bias measures reflect subject's willingness to report that a sensory attribute is present. For instance, while *C* = 0 refers to an unbiased response, a *C* > 0 indicates that the participant has a conservative response bias (i.e., higher likelihood of responding “The higher level of sweetness/fatness is not present” across all food samples). Conversely, a *C* < 0 indicates the participant has a liberal response bias (i.e., a bias toward responding “The higher level of sweetness/fatness is present” across all food samples). Finally, the second measure of response bias will be calculated in Excel as BETA = EXP((NORMSINV(F)^2^−NORMSINV(H)^2^)/2. Considering ß, the neutral point is ß = 1, while ß > 1 reflects a strict criterion (i.e., tendency to say “absent”) and ß < 1 reflects a lax criterion (i.e., tendency to say “present”).

In study 2, self-reports of pleasure, and facial expression responses will be characterized by a time-intensity (T-I) curve, and its constituent parameters, i.e., maximum intensity reached (Imax; 0–100), time elapsed to maximum intensity (Tmax; in seconds), and area under the curve (AUC; representing the overall emotion of the whole stimuli perceived over the total time of recording). Concerning heart rate variability, interbeat interval data will be exported to Kubios software (University of Eastern Finland, Kuopio, Finland). Inter-beat (R-R) time series will be corrected for artifacts, using a smooth priors de-trending method (λ = 500). Mean heart rate variability in terms of time and frequency domain parameters, mean skin conductance level and mean facial electromyographic amplitudes will determined before tasting food samples at baseline and thereafter.

#### Planned analysis

Analyses will be adjusted for covariates, namely the scores of EDE-Q, BDI-II, FCQ-T-Reduced, TSF-T-Reduced, and hunger and thirst levels. Additional covariates can be added to the model if necessary. In study 1, the procedure for computing the variance of d′, confidence intervals for d', chi-square tests of significance for d′ and comparisons of multiples d′s among groups across two testing conditions will be performed for sweetness, creaminess and caloric content scores according to the Bi et al. (1997) and Bi (2008) approach. Analogous to d′, confidence intervals, hypothesis tests and multiple comparisons for C and ß will be constructed according to Kadlec's (1999) approach.

In study 2, differences in T–I parameters (Imax, Tmax, AUC), self-ratings (hedonic, familiarity) and intensity of facial expressions (emotion, valence and actions units), as well as the cardiovascular, electrodermal and facial electromyographic outcomes, will be analyzed using mixed, three-way factorial ANOVAs with group (six levels) as between-subjects and testing condition (swallowing vs. sip-and-spit) and food sample (four levels) as within-subjects' factors. Additionally, a time series emotional analysis for assessing emotional responses to beverages will be applied to automated facial expression data using the Wilcoxon signed-rank test (Crist et al., 2016) if more sensitive analysis are needed to detect differences between groups. Outcome variables will be checked for normal distribution. Note that linear mixed models to analyse the data can be envisaged to have more statistical power to detect actually existing study effects and to lead to less biased results in the case that the intraclass correlation coefficient and the design effect show dependency in the data (Hox, 1998). Statistical analyses will be conducted using SPSS (IBM, SPSS; Version 23.0, Chicago, IL, USA). Descriptive statistics will be presented using Mean ± *SD* for all continuous variables.

### The expected results

Concerning the study 1, the main results to be expected are that a different and reduced C (*C* < 0) and (ß < 1) are expected in currently-ill AN and BN patients compared to non-ED underweight, normal-weight and illness-recovered controls, and during a swallowing compared with a sip-and-spit condition. Reduced C and ß-values would show a response bias to say, “Yes, the gaining weight-related attribute [sweetness, creaminess and caloric content] is present.” By contrast, patients will have a similar high d′ value compared with control groups, which means that all the participants have a good sensory discrimination.

Concerning the study 2, it is mainly predicted that restricting-type AN will show evidence of reduced verbal ratings as well as bodily higher corrugator activity, disgust facial expressions and heart rate acceleration in response to high-sweet and high-fat foods compared to stimuli of low-sweet and low-fat foods; non-ED underweight, normal-weight, illness-recovered controls and BN patients; and especially during the swallowing compared with the sip-and-spit condition. On the other hand, higher zygomatic muscle activity and more smiles expressions in response to very sweet tastes will be expected to observe in BN compared to fat and low-sweet stimuli; non-ED underweight, normal-weight, illness-recovered controls and restrictive-type AN; and no differences between swallowing and sip-and-spit conditions.

### Safety aspects

Adverse events are not expected and the planned measures for collecting personal data entail only minimal risks and burden. In particular, the present research collects data without using invasive behavioral or physiological procedures. Nevertheless, potential inconveniences due to using video recordings in this clinical sample will be properly addressed following current guidelines and recommendations (General Medical Council, 2002; Parry et al., 2016). As being video-recorded can raise anxieties and concerns and, therefore, can constitute an additional burden for patients, our research will provide several strategies ensuring video recordings are made in minimally intrusive ways: (1) we will reduce the intrusiveness of being filmed by using the laptop's inbuilt webcam, (2) researchers will not be in the same room during testing, and (3) a period of habituation to being filmed during the training session. For those participants who do not agree to be filmed, only facial electromyography recordings will provide measures of the hedonic reactions. Otherwise, hedonic reactions will be measured by facial expressions and facial electromyography only (to improve validity and reliability of hedonic assessment and enhance observations of small/subtle but meaningful emotional changes). Finally, during food tasting there will be more degustation than during a typical meal and thus the participants should consume only a small amount of chocolate ice cream in the swallowed condition, while they are requested to expectorate the food samples after assessing them in the spit condition. Doing so, this set-up is expected to reduce the inconveniences due to the fear of weight gain, especially in patients. Nevertheless, any aggravation of symptoms even when not related to the experimental procedure will be documented at every assessment throughout the study procedure. Finally, based on our previous experience with psychological and psychophysiological measures-related projects with EDs (e.g., Vögele and Florin, 1997; Vögele et al., 2009; Munsch, 2014), a framework for considering possible risks to participants' privacy, dignity and safety in all phases, and for planning measures that can reduce those risks is guaranteed.

## Discussion

Determining how food is actually perceived in patients with AN and BN is of crucial importance. First, if the current predictions are confirmed, restricting energy intake in AN might also be motivated by hedonic shifts, in which palatable food changes from a liked to a disliked or even disgusted entity. A better understanding of the conditions under which food becomes disgusting should help to explain why AN patients select and avoid certain foods and how they lose their appetite. Furthermore, additional knowledge on cognitive mechanisms in the top-down process of taste hedonics complements theoretical models and fosters the specificity of new cognitive interventions reducing taste aversions. Second, to the extent that taste aversions seem to be crucial in the trajectory and treatment of other forms of anorexia such as avoidant/restrictive food intake disorder (ARFID), vagotomy surgery- or cancer-induced anorexia (Bernstein and Borson, 1986; Hildebrandt et al., 2015), added knowledge on the development of aversions to dietary items might help to identify new targets in the prevention and treatment of at individuals at risk and people with AN or the taste characteristics of food-based ARFID (e.g., using flavor-pre-exposure procedures). Likewise, food learning techniques might be used for the acquisition of new functional food preferences (e.g., via conditioned-flavor learning) to overcome taste aversions in anorectics, and for the devaluation of the increased hedonic impact of sweet cravings and binge-eating in BN (e.g., via food-devaluation procedures). Third, exact knowledge about whether avoidance of high-caloric food is based on unpleasant taste responses or intentional actions to prevent weight gain, will shed light on previously neglected mechanisms related to the development of EDs. Moreover, future studies might investigate whether taste responsiveness represents a stable or variable marker of different types of EDs over time.

Interestingly, although dietary learning-based procedures have not been tested yet in patients with EDs, they have been successfully used in animal models and non-eating disordered human studies in an attempt to prevent, modify and reduce gustatory aversive associations and the hedonic impact of food stimuli. For instance, procedures to develop learned preferences for distasteful flavors are well-established in animal models (e.g., González et al., 2010). By contrast, well-known procedures that may reduce the effectiveness of and limit learning about the hedonic food reward concern flavor extinction (e.g., Garcia-Burgos and González, 2012), flavor pre-exposure (e.g., Garcia-Burgos et al., 2013), and blocking of flavor learning (e.g., González et al., 2014). In human studies, for instance, counter-conditioning and extinction interventions have been recently applied to target affective conditioning characteristic of disgust (e.g., Klucke et al., 2013; Hildebrandt et al., 2015). Moreover, food-devaluation procedures are currently being used to test behavioral sensitivity to reward in populations with weight problems (e.g., Horstmann et al., 2015). Therefore, evidence of disgust reactions in AN would raise some important possibilities for future research and development of clinical interventions, and the applications of dietary-learning for therapy appear to be promising as we move toward a more comprehensive understanding of the learning principles that affect EDs.

## Availability of data and materials

The dataset that will be analyzed during the current study will be available from the corresponding author on reasonable request. There will be no personal identification of participants in the data set.

## Author contributions

DG-B, SMu, and CV are responsible for the research design. DG-B drafted the paper, and all co-authors made significant contributions to drafting the protocol. All authors have read and approved the final manuscript.

### Conflict of interest statement

The authors declare that the research was conducted in the absence of any commercial or financial relationships that could be construed as a potential conflict of interest.

## Footnotes

^1^The terms hedonic, affective reaction, palatability, and liking are used interchangeably here to refer to the immediate pleasant/unpleasant experience produced when a substance is placed in the mouth.

## Abbreviations

AN: Anorexia Nervosa
ANOVA: analysis of variance
BDI-II: Beck Depression Inventory-II
BMI: Body Mass Index
BMS: Brief Mood Scale questionnaire
BN: Bulimia Nervosa
C-AN: currently ill anorectic
C-BN: currently ill bulimic
DIPS: Diagnostic interview for psychiatric disorders
ED: Eating Disorders
EDE-Q: Eating Disorder Examination Questionnaire
EMG: Electromyography
FCQ-T-reduced: Food Cravings Questionnaire—Trait
MSTL: Multiple-Sip Temporal-Liking
N-CT: normal weight control
R-AN: recovered anorectic
R-BN: recovered bulimic
SCID-I: Structured Clinical Interview for DSM-IV
SDT: Signal Detection Theory
T-I: Time-Intensity
TR: Taste Reactivity
TSF-B: Thought-Shape Fusion Body State Scale
TSF-Trait: Thought-Shape Fusion Trait Questionnaire
U-CT: underweight control.
